# To label or not: the need for validation in label-free imaging

**DOI:** 10.1117/1.JBO.29.S2.S22717

**Published:** 2024-12-20

**Authors:** Joseph M. Szulczewski, Filiz Yesilkoy, Tyler K. Ulland, Randy Bartels, Bryan A. Millis, Stephen A. Boppart, Kevin W. Eliceiri

**Affiliations:** a University of North Carolina-Chapel Hill, Department of Pharmacology, Chapel Hill, North Carolina, United States; b University of Wisconsin-Madison, Department of Biomedical Engineering, Madison, Wisconsin, United States; c University of Wisconsin-Madison, Department of Pathology and Laboratory Medicine, Madison, Wisconsin, United States; d Morgridge Institute for Research, Madison, Wisconsin, United States; e Vanderbilt University, Department of Biomedical Engineering, Nashville, Tennessee, United States; f Vanderbilt University, Vanderbilt Biophotonics Center, Nashville, Tennessee, United States; g University of Illinois Urbana-Champaign, Beckman Institute for Advanced Science and Technology, Urbana, Illinois, United States; h University of Wisconsin-Madison, Department of Medical Physics, Madison, Wisconsin, United States

**Keywords:** label-free imaging, autofluorescence, intrinsic contrast, probes, validation

## Abstract

**Significance:**

Advances in label-free imaging have impacted many areas of biological and biomedical imaging ranging from cell biology and cancer to pathology and neuroscience. Despite the great progress and advantages of these methods, it is clear that to realize their full potential, validation by extrinsic labels and probes is critically needed.

**Aim:**

This perspective calls for developing and applying innovative labels and probes to validate both existing and emerging label-free imaging methods.

**Approach:**

Major representative types of label-free imaging methods are briefly presented discussing their advantages and differing contrasts. Their biological applications are also reviewed with a focus on how validation of label-free methods with carefully developed labeling approaches will greatly aid in further intrinsic contrast imaging adoption and likely lead to more sophisticated image-based biomarkers and a better understanding of the underlying signals.

**Conclusions:**

Expanded efforts in extrinsic label validation will significantly push forward the utilization and adoption of label-free methods both in basic research and clinical models.

## 1 Introduction

It has been 30 years since Martin Chalfie first expressed green fluorescent protein in *Escherichia coli* and *Caenorhabditis elegans*.^1^ This event marked the beginning of a “fluorescence revolution” in biology, where advancements in microscopy and genetics allowed for the mapping of protein localization and direct observation of protein activity *in vivo*. During this time, microscopists made another observation that was originally deemed an experimental nuisance: “autofluorescence.” We now know that this background “noise” is the result of very specific intrinsic fluorophores and light scatterers, making this signal information rich. This endogenous fluorescence, together with other label-free imaging techniques, has become an exciting new feature in our imaging toolkit. Some of the prominent label-free imaging techniques include the following: (1) “autofluorescence” from tryptophan, an aromatic ring amino acid, used to study protein structure and function; metabolic co-factors nicotinamide adenine dinucleotide (phosphate) (NAD(P)H) and flavin adenine dinucleotide (FAD), proxies for cellular metabolism;^2^^,^^3^ lipofuscin;^4^ and elastin;^5^ (2) “second harmonic generation” (SHG) from non-linear scattering by collagen fibers has been widely utilized to observe the architecture of tissues and organs and has even been used to identify prognostic structural changes in disease;^6^^,^^7^ (3) “third harmonic generation” generated by intracellular lipid bodies helps decipherer their roles in inflammation and cancer;^8^ (4) “light scattering” within tissues can be quantified through techniques such as optical coherence tomography to reveal the structural components based on the differences in refractive indices, birefringence, and blood flow and can distinguish between live and dead tissues;^9^ and (5) “vibrational spectroscopies” (such as Raman and infrared) that facilitate label-free imaging of small molecules based on their characteristic vibrational spectra and elucidate important cellular processes including metabolism and lipid/protein content.^10^^–^^12^

Utilizing label-free imaging techniques to observe intrinsic signals provides numerous significant advantages, both compared with and in combination with exogenous labels, ranging from applications in fundamental biomedical research to the clinical laboratory. In the biomedical research space, countless research dollars and time (in some cases a year or more) may be dedicated to the integration of exogenous labels into model systems of interest, even after refined probes have been developed (which, in some cases, may take years to develop themselves). In many cases, ideal model systems for a particular research project are abandoned at the start due to the lack of genetic tools to engage in such labeling. Although sophisticated probes are regularly being developed and are an essential part of biomedical research, their requisite irradiance when compared with certain unlabeled approaches (quantitative phase or polarization, for example) phototoxicity of the specimen remains a significant concern. Further, one must be vigilant about the many ways artifacts can be introduced by genetic manipulation, overexpression, and/or chimeric nature that many transgenic approaches may present. Clinical use of imaging approaches with exogenous labels is often a non-starter due to safety concerns and potential side effects. Thus, fewer options exist in this space for thorough evaluation of otherwise promising diagnostic or therapeutic methodologies. In addition, certain intrinsic imaging strategies report quantitative information, such as chemically specific spatiotemporal contrast, that currently cannot be examined adequately by synthetic probes. Notably, vibrational spectroscopy can provide complex spectral data that contain information about molecular functional groups and overall symmetry, which cannot be captured by labels. Although certain unlabeled approaches traditionally suffer from weaker signals than their labeled counterparts, newer approaches hold great promise in improving upon this. For example, quantitative polarization-based imaging can serve as a functional reporter and proxy for critical cellular attributes, such as biomechanical force (contractility), in natural and diseased states.^13^ Such attributes have vital implications in the context of wound healing, development, and invasive cancers, to name a few. In addition, other methods such as quantitative phase imaging (QPI)^14^^,^^15^ combine the benefits of examining morphology by microscopy with additional contrast by quantifying the changes in the phase shift as light passes through a sample. QPI has been widely used in diverse applications ranging from pathology and cancer^16^^,^^17^ to cell biology^18^ and neuroscience.^19^ The effectiveness of all these methods in real-world biomedical research and clinical applications depends on our ability to accurately interpret the data produced, especially in complex biological contexts. This underscores the importance of correlative studies that provide reliable biological ground truth, helping us better understand the complex intrinsic signals from label-free imaging techniques. From this perspective, we aim to highlight a few areas where the development of new sensors and probes could provide further validation of label-free imaging techniques.

## 2 Autofluorescence

### 2.1 NADH Fluorescence and Metabolism

Imaging endogenous fluorescence of NAD(P)H and FAD has shown enormous promise in providing metabolic readouts *in vivo*.^3^^,^^20^^,^^21^ Taking advantage of the fact that NAD(P)H and FAD are autofluorescent, their oxidized or reduced counterparts [NAD(P)^+^ or ${\mathrm{FADH}}_{2}$] are not, allowing for the calculation of optical redox ratios, which represent the state of cellular metabolism at subcellular scales. Although varying definitions of redox ratio have been used, largely based on the pioneering work of Chance et al.,^22^ the optical redox ratio has been widely defined as the intensity of NADH divided by the intensity of FAD.^23^ With the development of fluorescence lifetime imaging microscopy (FLIM),^24^ the utility of NADH endogenous fluorescence has been further extended with FLIM able to measure glycolysis via the discrimination of bound versus free NADH.^25^^–^^27^ Whether based on intrinsic fluorescence alone or via FLIM measurements, such capability of detecting spatial and temporal metabolic contrast has contributed to many areas of biology, including stem cell differentiation,^28^ cancer,^3^ and its interplay with the immune system,^29^ including microglia metabolism.^30^ However, this technique has its limitations, as the NAD(P)H autofluorescence signal is indistinguishable between the relative contributions of NAD(P)H and NADH, both of which are metabolically important, thus obstructing definitive interpretation of these readouts. Traditional methods to determine NADH and NAD(P)H concentrations require the excision and disassociation of tissue, which destroy spatial information and leave cells potentially prone to oxidation. Oxidation is problematic because it modifies the innate molecular states within the cell, distorting the interpretation of the data. Therefore, autofluorescence imaging would greatly benefit from well-designed biosensors that could alleviate these concerns and serve as very useful validation.

Luckily, several biosensors have recently been developed that could address many of these issues. Traditional metabolic sensors based on ribonucleic acid (RNA) and transcription factors can generate readouts to different metabolic processes, oxidative phosphorylation, and lipid biogenesis. However, these genetic readouts rely on mechanisms of transcription and translation and often lag minutes to hours behind the initial metabolic changes. Other techniques based on “protein activity,” such as ones that utilize fluorescence resonance energy transfer^31^ or circularly permuted fluorescent proteins,^32^ have readout times closer to milliseconds to seconds, providing more informative temporal data. There are a few recently reported biosensors that are attempting to differentiate NADH and NAD(P)H: sensor of NAD(H) redox (SoNar) biosensors have been designed to detect NADH/NAD^+^-specific ratios and are based on circularly permuted yellow fluorescent protein.^33^ Moreover, NADP^+^ levels have been characterized with anisotropy Apollo-NADP^+^ sensor, and a new NAD(P)H-estimating ratiometric non-destructive sensing tool has been reported to show NAD(P)H/NADP^+^ redox ratio through fluorescent readout, which are exciting validation tools to help advance NADH endogenous fluorescence.^34^ However, limitations still exist because these sensors are sensitive to pH and temperature changes and often contain fluorophores with significant spectral overlap with NAD(P)H emission. One must consider the potential challenges in separating signals from autofluorescence and fluorescent dyes/probes, particularly when their spectral characteristics overlap. To address this issue, a combination of quantitative approaches can be employed, including FLIM and spectral deconvolution techniques. FLIM utilizes the distinct fluorescence decay profiles of different fluorophores and autofluorescent species, providing an additional layer of specificity. Spectral deconvolution can further enhance signal separation by analyzing the contributions of individual components across the emission spectrum. Together, these approaches enable robust discrimination of overlapping signals.

### 2.2 Autofluorescence for Cell Type Identification

To fully harness the potential of autofluorescence, particularly in complex biological environments, there is a critical need for highly specific label-based methods. These methods are essential for advancing, defining, and validating label-free approaches by providing a reliable reference for accurate cell identification and metabolic profiling. There is a great promise that NAD(P)H and FAD^+^ autofluorescence could be used to provide cell-specific metabolic contrast in a variety of different contexts. Changes in^35^ NADH and FAD^+^ autofluorescence have been used to monitor stem cell differentiation and macrophage and microglia activation *in vivo*. In fact, the metabolic characterization of cells, such as microglia and macrophages, in their three-dimensional local cellular environment, is perhaps one of the most exciting applications of endogenous fluorescence imaging. For example, the ability to identify and metabolically profile microglia in the brain during neurodegenerative pathology, such as Alzheimer’s disease, has been an aspiration in the biophotonics field.^36^ The use of label-free fluorescent microscopy can facilitate a more granular dissection of the function of plaque-adjacent microglia, elucidating their previously unknown metabolic profiles and role in disease progression. In breast cancer, NADH and FAD^+^ autofluorescence can be used to distinguish macrophages from cancer cells in the breast tumor microenvironment.^21^ Moreover, the optical redox ratio can reveal the effectiveness of chemotherapeutic treatment^37^ and add context to the importance of tumor-associated macrophage metabolism. Although these techniques show great promise in providing label-free cellular contrast *in vivo* for both microglia and macrophages, there is still a significant need for new probes that can be used in concert with these techniques to further enhance the diagnostic value of these observations. For example, current means for labeling cell types require the staining of fixed tissues, which loses dynamic context and destroys some metabolic information. Other alternatives are the genetic expression of fluorescent proteins based on cell type-specific promotors along with the creation of mouse models, which are often cost-prohibitive and can be complicated in model systems with limited genetic manipulability. In addition, dye-labeled antibodies specific to cell surface markers have been used,^21^ but these have limitations in depth of diffusion in *in vivo* applications, restricting accurate identification to just the most superficial contexts. Notably, due to their decreased size over traditional monoclonal antibodies, dye-labeled nanobodies specific to cell type receptors could aid in the identification of deeper cells.^38^ Ultimately, the development of probes that easily diffuse through tissues and only get fluorescently activated when bound to specific cell surface receptors would push this area of exploration further. These probes would provide an invaluable biological ground truth to inform endogenous fluorescence and aid in its ability to answer specific biological questions by identifying specific cell types and their activation states.

### 2.3 Label-Free Raman and Infrared Vibrational Spectroscopies

One of the key goals of optical microscopy in biology is to observe how biomolecules behave and interact in real time. Vibrational spectroscopy offers an exciting, label-free way to image the chemical makeup of cells. Although it is widely used in chemistry to identify substances, applying it to live-cell imaging is still a challenge. So far, it is mainly been used to detect broad categories of molecules such as lipids, nucleic acids, proteins, carotenoids, and porphyrin-containing proteins.^39^^–^^41^

The difficulty lies in the nature of vibrational spectroscopy, which includes techniques such as Raman and mid-infrared (MIR) spectroscopy.^41^ Raman spectroscopy is limited by its extremely weak signal in which only 20 photons would be collected after a 10-s exposure. In contrast, fluorescent molecules absorb and emit light much more efficiently ∼10 trillion times stronger than Raman scattering. Under the same conditions, fluorescence can collect around >150 photons in just 1  μs, easily detecting individual molecules in real time. Such a weak signal limits both imaging speed and sensitivity to detecting changes in molecular constituents. Non-linear optical interactions that stimulate coherent Raman scattering (CRS) have been exploited to boost Raman imaging speed by $>10^{6}$ times—allowing video-rate CRS imaging of cells.^42^^,^^43^ Despite these challenges, Raman microscopy has been shown to provide valuable information for stem cells and embryogenesis.^44^

Although Raman spectroscopy is an impactful tool for specific applications, and highly specific, it is not yet widespread as a biomedical research tool, given its limited analytical performance in complex biosamples. Raman microscopy is an area where a continued community-driven effort to validate constituent signals amongst complex biological contexts could unlock significant gains in the application of unlabeled technologies.

MIR vibrational spectroscopy exploits dipole-allowed optical transitions, resulting in an optical response only 100 times weaker fluorescence.^45^^,^^46^ However, MIR absorption spectroscopy comes with restraining challenges. First, the wavelength of infrared radiation in the MIR spectral range is >10× longer than visible wavelengths, which leads to poor spatial resolution, and is generally incapable of sub-cellular scale. Second, water is a strong and broad absorber in the MIR spectrum, which interferes with the molecular absorption signatures, hindering the ability for live cell analysis and deep tissue imaging.

The potential power of vibrational spectroscopic imaging motivates the development of new spectroscopic imaging methods to improve the speed and sensitivity of chemical imaging. Recent strategies were adopted to improve imaging speed for Raman spectroscopy using high-speed optical delay lines,^47^^,^^48^ computational imaging,^49^^,^^50^ and widefield imaging.^51^ Efforts to improve sensitivity in Raman detection have made use of novel detection strategies,^52^^–^^54^ rear-resonant enhancement,^55^^,^^56^ and photothermal detection^57^^,^^58^ In addition, developments pushing technology for low-frequency Raman spectroscopy (with vibrational frequencies below $200\;{\mathrm{cm}}^{-1}$) offer the potential for studying mechanical soft materials such as biological tissues in a non-invasive manner.^54^

Technological developments in infrared photonics have also advanced MIR spectrochemical detection. For example, efforts have focused on MIR signal enhancement with metasurfaces,^59^ MIR photothermal imaging,^60^^,^^61^ and combined MIR and fluorescent detection^62^ that allows for rapid imaging in live cells with visible or near-infrared detection—vastly improving spatial resolution. Future work with these new chemical imaging modalities will reveal the extent to which these recent advances expand our routine toolsets used for biological studies.

Vibrational spectroscopy has been applied to numerous biological systems, facilitating spatially resolved molecular fingerprint chemical imaging. However, the complexity of molecular composition in biological systems makes the interpretation of specific biomolecular interactions difficult to discern. This challenge lies in the fact that the molecular building blocks for biological macromolecules use a limited pallet for critical components such as nucleic acids, proteins, and lipids. Thus, it is critically important for the community to establish standards for chemical imaging through Raman or MIR spectroscopic measurements and validate the molecular information retrieved from the spectrochemical image datasets.

Here too is a place where fluorescent labels shine through with powerful capabilities because specificity in the labeling of molecules of interest facilitates detailed and specific studies. The combined use of fluorescent labels with Raman and MIR imaging could help establish the utility of chemical imaging in real-world biomedical applications. In addition, information from fluorescent labels could reveal additional ground truth information that, when coupled with spectroscopic imaging, could help test and validate unique molecular fingerprints in spectral signal analysis. Moreover, the fluorescent images can be used as ground-truth labels in supervised machine learning (ML) models, enabling the retrieval of quantitative and molecular-specific information from vibrational spectroscopic image datasets. Such capabilities will greatly benefit from sensitive spectral measurements capable of capturing accurate molecular information, as well as large datasets for reliable and unbiased training, testing, and validation of the ML models. As these ML models rely on large datasets, improved imaging speed is of great importance for various applications, such as high throughput screening (e.g., drug responses to patient-derived cells), whole-slide tissue imaging, flow cytometry, or studying the chemical processes of rapidly moving inter- and intra-cellular components such as exosomes and organelles.

In applications where fluorescent labels fall short in providing the specific molecular information to cross-correlate with the vibrational spectral data, multimodal imaging approaches can be implemented.^63^^–^^65^ For example, in a recent work, second harmonic generation imaging was used in tandem with MIR chemical imaging to study tumor microenvironment in pancreatic tissue samples.^63^ In this study, the unique specificity of SHG microscopy to fibrillar collagen was leveraged using the SHG images as ground-truth collagen labels in an ML model to identify spectral signatures of fibrous tissue regions. Such multimodal imaging investigations can help elucidate the chemical alterations that lead to the architectural changes in tissues associated with various pathologies. Similar label-free multimodal approaches can be used between the vibrational spectroscopies and multiphoton metabolic imaging techniques to decode the spectral cues associated with the metabolic states of cells.

The inherent differences in spatial resolution between fluorescence-based imaging techniques and vibrational spectroscopies, such as Raman or infrared, present unique challenges. For scenarios requiring higher spatial resolution or validation of subcellular signal sources, super-resolution microscopy or expansion microscopy offers complementary solutions. Expansion microscopy, in particular, enables physical magnification of the sample, facilitating the co-registration of spatially resolved fluorescence and vibrational data.

## 3 Concluding Remarks

Label-free imaging techniques hold immense potential in the biological and medical sciences. They enable non-destructive and quantitative analysis of biological systems and may stand to accelerate discovery by relieving investigators of the myriad inefficiencies of creating labeled specimens or teasing apart the artifacts of doing so. Such luxuries do currently come at significant cost. Although these differ depending on the approach, overcoming the current limitations of many share a need for careful discernment, and thus, validation of the rich, but often convoluted, signatures we collect. In the case of autofluorescent readouts of metabolic proxies, for example, having a more granular level understanding of what is being reported by overlapping AF spectra between metabolic constituents with distinct roles, would greatly inform studies spanning many research interests. Spectral signals mapped across complex biospecimens are often congested, and vastly more so should dynamic temporal signatures be required. In addition, the quantitative and qualitative molecular perturbations associated with malignancies and other pathology can be subtle, requiring highly sensitive and high-throughput optical measurements. Significant efforts have been devoted to enhancing the analytical sensitivity, SNR, and signal acquisition speed in optical imaging and spectroscopy measurements. In this perspective, we have highlighted the critical importance of cross-validating label-free imaging data with well-established imaging modalities that offer molecular specificity. Such validation efforts, using deterministic experimental protocols on simple model systems, can help demonstrate the utility of chemical imaging and unleash the full potential of label-free methods as powerful next-generation bioanalytical tools. There is great potential for other approaches that could be used to help validate a particular label-free approach such as other already-validated label-free imaging methods and deep learning, but specific extrinsic labels will always play a clear role in understanding label-free contrast.

## Data Availability

Data sharing is not applicable to this paper, as no new data were created or analyzed.
